# Effectiveness of botulinum toxin injection in the treatment of de novo OAB symptoms following midurethral sling surgery

**DOI:** 10.1007/s00192-015-2839-x

**Published:** 2015-09-12

**Authors:** Pawel Miotla, Konrad Futyma, Rufus Cartwright, Michal Bogusiewicz, Katarzyna Skorupska, Ewa Markut-Miotla, Tomasz Rechberger

**Affiliations:** 2nd Department of Gynaecology, Medical University of Lublin, ul. Jaczewskiego 8, 20-954 Lublin, Poland; Department of Epidemiology and Biostatistics; Department of Urogynaecology, Imperial College London, London, UK; Department of Paediatric Nursing, Medical University of Lublin, Lublin, Poland

**Keywords:** Botulinum toxin, De novo OAB, Midurethral sling, Overactive bladder, Quality of life, Urgency incontinence

## Abstract

**Introduction and hypothesis:**

Intravesical onabotulinumtoxinA (Botox®) is effective for idiopathic overactive bladder (OAB) symptoms. Our primary objective was to compare the efficacy of onabotulinumtoxinA for women with de novo OAB after midurethral sling (MUS) surgery and women with idiopathic OAB.

**Methods:**

Women enrolled in this prospective study had idiopathic (*n* = 53) or de novo (*n* = 49) OAB symptoms after MUS, with at least one episode of urgency urine incontinence per day. OnabotulinumtoxinA (100 U) was administered in 20 intradetrusor injections. Postvoid residual volumes were checked at 2, 4 and 12 weeks. Participants completed a 3-day bladder diary and the King’s Health Questionnaire (KHQ) before and 12 weeks after treatment.

**Results:**

After 12 weeks, 22 patients (41.5 %) in the idiopathic OAB and 19 patients (38.8 %) in the de novo OAB groups were completely dry. OnabotulinumtoxinA injections had a significant benefit within both groups (*p* <0.001) to decrease both the daily numbers of voids (−2.39 and −2.0) and incontinence episodes (−1.38 and −1.44), with no significant difference between groups. We observed an increase of mean voided volume of >90 ml in both groups. Urinary retention was observed in four patients.

**Conclusions:**

We observed similar improvement in OAB symptoms after intravesical onabotulinumtoxinA injections within both groups. The rates of retention and requirement for catheterization even for women with a prior MUS were acceptable. These observational data provide evidence that onabotulinumtoxinA can effectively treat patients with OAB following stress urinary incontinence surgery.

## Introduction

Midurethral slings (MUS) are widely used as first-line surgical therapy for women with stress urinary incontinence (SUI). Rates of surgery have sharply increased over the last decade [1], with a recent estimate for the lifetime risk of SUI surgery of 13.6 % [2]. With current demographic trends, it is expected that the number of MUS surgeries performed will continue to increase.

Overall, these minimally invasive procedures have a low incidence of complications. It was described that similarly to earlier procedures for SUI—e.g., Burch colposuspension—insertion of an MUS may also be associated with the development of urge urinary incontinence (UUI) and storage-related lower urinary tract symptoms (LUTS). Reports indicate that 6–8 % of women treated with MUS will develop de novo overactive bladder (OAB) symptoms [3, 4].

New-onset storage LUTS significantly impact on subjective cure rates and patients’ satisfaction with these procedures [5]. The mechanisms leading to symptoms of de novo OAB are still not well understood but may occur even after a first MUS surgery with correct tape placement [6]. In some cases, OAB symptoms may be attributed to excessive tension applied to the tape or its placement too close to the bladder neck [7–9]. After tape malposition, patients are likely to benefit from tape incision; however, most women will require pharmacological treatment. There are limited data regarding the efficacy of therapies, specifically in patients with de novo OAB after MUS.

Symptoms of idiopathic overactive bladder (OAB) affect ∼17 % of women, and its prevalence increases with patient age, achieving 30.9 % in elderly patients [10]. One of the most bothersome symptoms of OAB is UUI, followed by urgency and frequency [11]. The standard pharmacological treatment for idiopathic OAB starts with anticholinergic drugs or mirabegron intake [12, 13]. Many patients with idiopathic OAB cannot be adequately managed with orally administered drugs. Recent evidence also suggests that antimuscarinic therapy provides less subjective benefit for patients with de novo compared with idiopathic OAB [14].

Intravesical injections of onabotulinumtoxinA or sacral nerve stimulation are widely used third-line options for OAB. The efficacy of onabotulinumtoxinA (100 U) in the treatment of idiopathic OAB has been repeatedly shown in randomized clinical trials (RCTs) [15, 16]. Compared with placebo, it significantly decreases daily micturition episodes, and the number of UUI episodes, and importantly also significantly improves health-related quality of life in patients with idiopathic OAB [15–18]. However, no studies have tested the benefits of onabotulinumtoxinA specifically for patients with de novo OAB after MUS procedures. Hence, we designed a study aiming to compare the efficacy and safety of intravesical onabotulinumtoxinA injections in women with de novo and idiopathic OAB. The primary endpoints were changes in daily number of micturitions and UUI episodes from baseline to week 12. Secondary endpoints were changes in voided volume, differences in pad use, and changes in King’s Health Questionnaire (KHQ) scores compared with baseline. Safety assessments included all common potential adverse events of botulinum toxin, including urinary retention, increased postvoid residual (PVR) volumes, and urinary tract infection (UTI).

## Materials and methods

The study protocol was approved by our Institutional Board Review for the Medical University of Lublin, Poland. All participating patients were counselled regarding potential adverse effects of onabotulinumtoxinA; they gave written informed consent. Inclusion criteria were as follows: nonpregnant women >18 years of age; idiopathic OAB wet symptoms or de novo OAB wet symptoms (at least one urine leakage per day and more than eight micturition daily); lack of efficacy (at least 3 months) or intolerance to antimuscarinic therapy; stage 0 or 1 on the Pelvic Organ Prolapse Quantification (POP-Q) scale.

Exclusion criteria were mixed urinary incontinence (MUI) in the past; OAB symptoms before sling surgery; bladder-outlet obstruction (BOO) after surgery [maximum flow (Q_max_) on uroflowmetry < 15 ml/s]; UTI; neurologic disorders affecting bladder function; PVR volume >100 ml; contraindications to onabotulinumtoxinA use; allergy to lidocaine; uncontrolled systemic diseases; contraindications to clean intermittent self-catheterization; previous prolapse or anti-incontinence surgery (except midurethral sling surgery with transobturator approach; only one procedure in the past and at least 6 months before onabotulinumtoxinA treatment); previous onabotulinumtoxinA injections due to urological conditions. All participants were of European descent. From November 2011 to June 2014, 53 patients with idiopathic and 49 with de novo OAB after MUS surgery were enrolled. All women in the MUS group had been treated with transobturator tapes (T-sling, Polhernia) and were enrolled in our study a minimum of 6 months after that surgery. At enrollment, all patients had been treated for at least 3 months with anticholinergics—either without sufficient improvement in OAB symptoms, or with treatment limited by adverse events.

Participants completed 3-day bladder diaries, including a count of pads used. They also completed a validated translation of the KHQ for evaluation of incontinence-specific quality of life (QoL) impairment, and they finally underwent a systematic clinical examination. Postvoid residual measurements using abdominal ultrasound (US) were performed in all patients before treatment. Urodynamic testing was assessed to confirm OAB symptoms and exclude the possibility of BOO and therefore the need for sling incision. Additionally for the sling group, transvaginal US was performed as described previously in order to exclude patients with tape malposition who could benefit from sling incision [8, 9]. Two blinded evaluators reviewed outcome data before analyses.

Participants started with antibiotic prophylaxis (ciprofloxacin 500 mg per os twice daily) for 1 day before the injection procedure and continued for 5 days after treatment. Thirty minutes before the procedure, all patients received a 100-ml instillation of 2 % lidocaine intravesically through a Foley catheter. Saline cystoscopy was performed using a 19-F rigid cystoscope (Karl-Storz). OnabotulinumtoxinA (100 U) in 10 ml 0.9 % sodium chloride was administered in 20 injections during cystoscopy, sparing the trigone, using a 25-gauge injeTAK 198 DIS needle (Laborie). The tip of needle was inserted 3 mm into the bladder wall. Patients were discharged 2–3 h after injections and following a successful demonstration of voiding. PVR volumes were checked after 2 weeks, 1 month, and 3 months. Patients with elevated residual volumes (PVR > 100 ml) received extended antibiotic prophylaxis (e.g., nitrofurantoin 100 mg three times per day). Patients completed a further bladder diary and the KHQ after 3 months.

Statistical analysis was performed with Statistica Statsoft, version 10 package, using the unpaired or paired Student *t* test, the Mann–Whitney *U* test, and the χ2 test, as appropriate. A *p* value < 0.05 was considered statistically significant throughout. The sample size was pragmatic, but post hoc power calculations based on the observed success rate, taking into account the decrease in mean number of incontinence episodes and frequency of micturitions as primary endpoints and using the *t* test for paired samples, showed >95 % power at a two-sided significance level of 0.05 for each group.

## Results

### Patient demographics

**Fig. 1:**
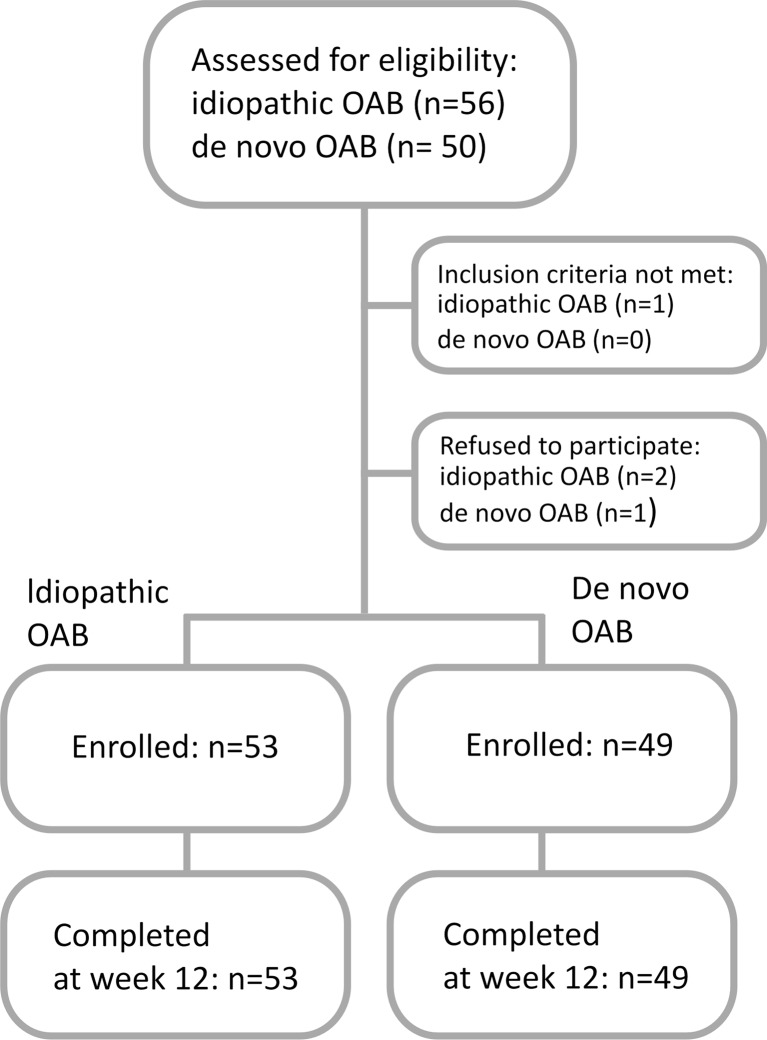
Study participants

Baseline demographic characteristics were similar between groups, with the exception of higher parity among women with a prior MUS (Table 1). One hundred and two patients completed treatment and were available with 12 weeks of follow-up (Fig. 1).

**Table 1:** Demographic characteristics of patient groups

Variable	Idiopathic OAB (*n* = 53)	De novo OAB after MUS (*n* = 49)	*p* value
Age (years)	58.7 ± 15.3	63.6 ± 10.2	0.06
BMI (kg/m^2^)	29.1 ± 5.3	30.1 ± 5.0	0.12
Parity	1.01 ± 1.53	1.75 ± 2.53	<0.001
Previous hysterectomy	11 (20.75)	7 (14.3)	0.39

Continuous variables are mean ± standard deviation; categorical variables are *n* (%)

*BMI* body mass index, *OAB* overactive bladder, *MUS* midurethral sling

### Clinical effectiveness

**Fig. 2:**
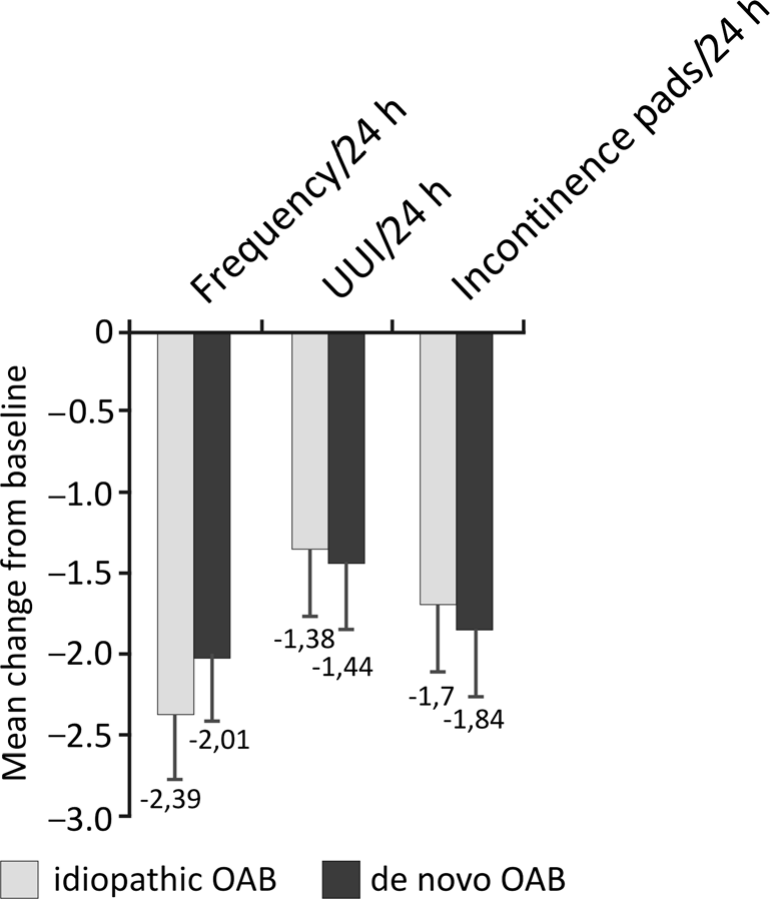
Change from baseline in number of daily micturitions, urge incontinence episodes, and pads used in patients with idiopathic and de novo overactive bladder (OAB) at week 12 after onabotulinumtoxinA injections. There were no statistically significant differences between idiopathic vs de novo OAB groups

**Fig. 3:**
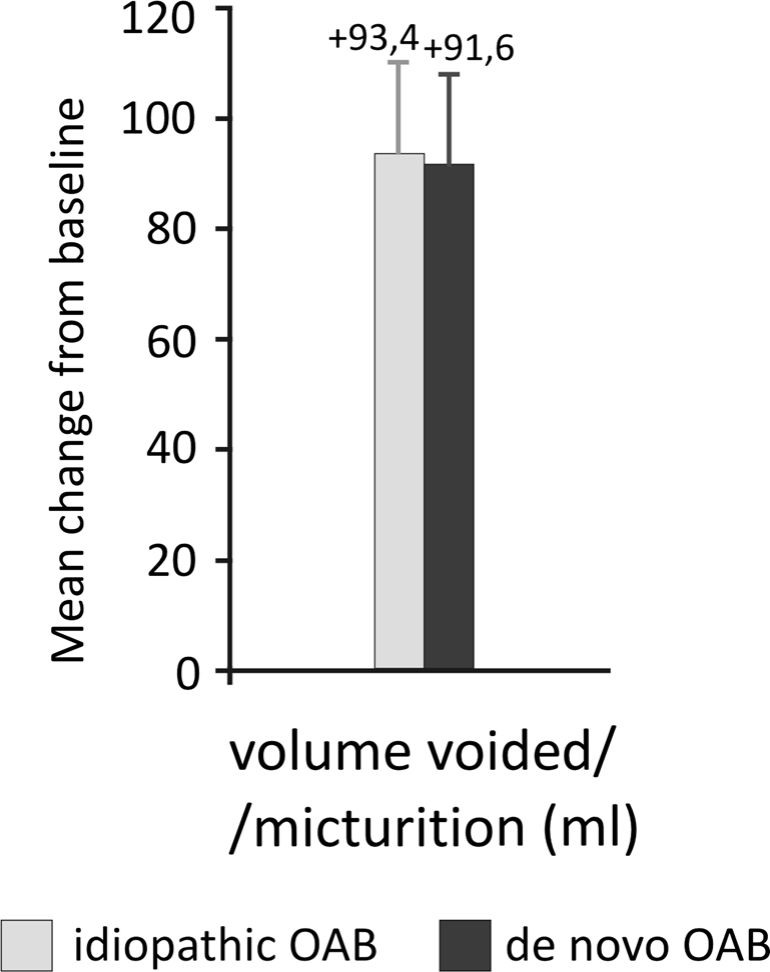
Change from baseline in voided volume in patients with idiopathic and de novo overactive bladder (OAB) at week 12 after onabotulinumtoxinA injections. There was no statistically significant difference between idiopathic and de novo OAB groups

After 12 weeks, 22 (41.5 %) patients with idiopathic and 19 (38.8 %) with de novo OAB were completely dry on their 3-day bladder diaries, and the rates of dryness did not differ between groups (*p* > 0.05). Taking into account that all these patients had at least one episode of urine leakage per day at baseline, these data support high effectiveness of the procedure. OnabotulinumtoxinA injections significantly decreased the mean number of daily micturitions, incontinence episodes, and number of pads used, as well as increasing mean voided volume per micturition in both groups. Across all outcomes, efficacy was similar for patients with idiopathic and de novo OAB, with no statistically significant differences between groups (Table 2; Figs. 2 and 3).

**Table 2:** Pre- and posttreatment clinical parameters in patients with idiopathic and de novo overactive bladder (OAB) who received intravesical onabotulinumtoxinA injections

Parameters	Idiopathic OAB (*n* = 53)	De novo OAB after MUS (*n* = 49)
Number of micturitions/24 h (pretreatment)	10.89 ± 1.43	10.94 ± 1.34
Number of micturitions/24 h (posttreatment)	8.50 ± 2.05	8.93 ± 1.84
Number of UUI/24 h (pretreatment)	1.87 ± 0.86	2.12 ± 0.86
Number of UUI/24 h (posttreatment)	0.49 ± 0.65	0.68 ± 0.94
Volume voided (ml)/micturition (pretreatment)	202.3 ± 30.0	199.0 ± 35.7
Volume voided (ml)/micturition (posttreatment)	295.7 ± 62.1	290.6 ± 77.9
Number of incontinence pads used/24 h (pretreatment)	3.07 ± 0.97	3.47 ± 1.32
Number of incontinence pads used/24 h (posttreatment)	1.37 ± 0.88	1.63 ± 1.25

All data presented as mean ± standard deviation. Differences between pre- and posttreatment values within each group were statistically significant (all *p* < 0.001). There were no statistically significant differences between groups

### Quality of life

**Fig. 4:**
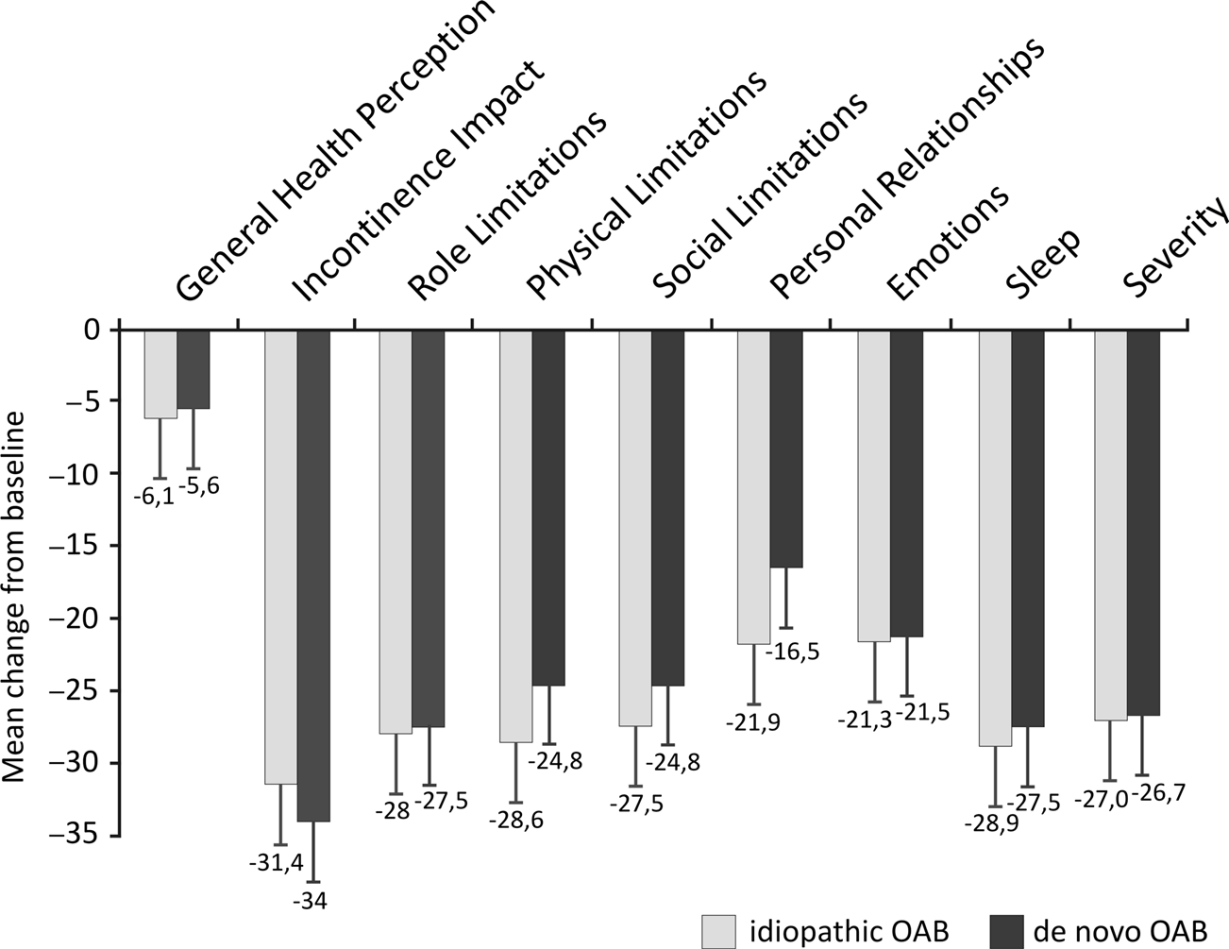
Change from baseline in quality of life in the King’s Health Questionnaire at week 12 after onabotulinumtoxinA injections. Differences between pre- and posttreatment values in each investigated group were highly statistically significant (all *p* < 0.001). There were no statistically significant differences between groups

At the 12-week follow-up, participants in both groups reported lower values in all questionnaire domains compared with baseline (high KHQ scores represent greater QoL impairment). There were statistically significantly improvements at 3 months in each domain in both groups (*p* < 0.001), including the General Health Perception domain. We found no statistically significant differences in QoL improvement between groups either for individual domains or for total KHQ score (Fig. 4).

### Safety

The most frequently observed adverse event in both groups was the increase in residual volume, which was usually asymptomatic and clinically nonsignificant. Elevated residual volumes were most prevalent during the US examination performed 2 weeks after treatment. The number of patients with elevated PVR volumes decreased from week 2 to week 12 in both groups. The most severe adverse event was urinary retention, which was observed in four patients (one in the idiopathic group with a postvoid volume of 300 ml, and three in the de novo OAB, two of whom had PVR > 350 ml and one 310 ml); two cases in the de novo group required self-catheterization for 1 month. In the other two patients, PVR decreased to 250 ml and 280 ml after one week, respectively. Urinary tract infections were rarely observed, at less than 4 % in both groups. We did not observe any major complications, requiring readmission or reoperation, in either group (Table 3).

**Table 3:** Incidence of adverse events at weeks 2 and 12 after onabotulinumtoxinA injections

Adverse event	Idiopathic OAB (*n* = 53) (at week 2)	Idiopathic OAB (*n* = 53) (at week 12)	De novo OAB after MUS (*n* = 49) (at week 2)	De novo OAB after MUS (*n* = 49) (at week 12)
Urinary tract infection	2 (3.7)	1 (1.8)	2 (4.0)	1 (2.0)
Hematuria and/or leukocyturia	1 (1.8)	0 (0)	3 (6.1)	1 (2.0)
Weakness and fatigue	0 (0)	0 (0)	1 (2.0)	0 (0)
Residual urine volume <100 ml	20 (37.7)	5 (9.4)	13 (26.5)	7 (14.2)
Residual urine volume >100 ml and <200 ml	7 (13.2)	2 (3.7)	12 (24.5)	3 (6.1)
Residual urine volume >200 ml and <350 ml	1 (1.9)	0 (0)	1 (2.0)	0 (0)
Urine retention >350 ml	0 (0)	0 (0)	2 (4.0)	0 (0)

Differences between investigated groups were not statistically significant. Data presented as *n* (%)

*OAB* overactive bladder, *MUS* midurethral sling

## Discussion

We believe this study is the first to evaluate the effectiveness of onabotulinumtoxinA specifically for patients with de novo OAB symptoms after MUS surgery. Our results demonstrate that the efficacy of onabotulinumtoxinA injections is comparable with efficacy for idiopathic OAB, providing tape malposition is excluded. The primary endpoint of complete dryness measured at 12 weeks, observed in almost 39 % of de novo OAB patients, demonstrated a high cure rate for UUI, with clinically relevant and statistically significant improvements in all secondary outcomes.

Women in the de novo group reported reductions in both the daily number of UUI episodes and the frequency of voiding, with an increase in voided volume >90 ml. These objective findings are similar to data from recent randomized trials of onabotulinumtoxinA (100 U) injections in patients with idiopathic OAB [15, 18]. We also observed improvement in all domains of the KHQ in both groups, and again our results are consistent with published observations from large RCTs investigating idiopathic OAB [15, 16].

We observed UTIs in 4 % of patients only, a rate considerably lower than in previous clinical trials [15]. The incidence of elevated PVR volumes was similar to results of previous trials [15, 16]. Therefore, the low incidence of UTI may be attributable to our routine use of antimicrobial prophylaxis before and after cystoscopy and use of prophylaxis for all patients with elevated PVR volumes.

We found only one previous comparative study assessing the efficacy of pharmacological therapy in women with de novo (*n* = 110) and idiopathic (*n* = 120) OAB [14]. Patients in both groups were treated with 5 mg of solifenacin taken orally once daily for 3 months. In that prior study, patients in the de novo group reported significantly worse subjective and objective results after 3 months. More effective treatment options are needed for patients with de novo OAB symptoms, and overall, our results confirm the efficacy and safety of onabotulinumtoxinA in this setting.

The strengths of our study include the excellent follow -up achieved in a highly pragmatic and generalizable setting. Limitations include the relatively small number of patients and lack of placebo control. Lack of statistical significance for comparisons between groups must therefore be interpreted with caution considering the number of participants. Our findings may be subject to bias from unmeasured confounding, necessitating future randomized trials.

We observed that onabotulinumtoxinA injection was both effective and safe for patients with de novo OAB after MUS surgery, with comparable results to women with idiopathic OAB. Injections increased voided volume, decreased urinary frequency and number of UUI episodes, and improved QoL. Although the study provides limited power to test differences in adverse events between groups, the rates of retention and requirement for catheterization even among women with a prior midurethral sling were acceptable. Patients with de novo OAB must be warned about the potential increased risk of urinary retention. These observational data provide evidence that onabotulinumtoxinA injections can effectively treat patients with de novo OAB following incontinence surgery.
